# Foreign

**Published:** 1841-11-13

**Authors:** 


### FOREIGN.

*Remarks on Partial Fracture of the Radius.* By GIDEON ALGERNON MANTEL, LL.D., F. R. S., etc.—In the admirable lectures on surgery by M. Phillips, it is stated that a fracture may be incomplete, although some surgeons have denied the possibility of the occurrence; and, as conclusive of the fact, a sketch is given of a bone which had sustained such an injury.

Six cases of this kind have occurred in my practice during the last twenty-five years; and as the diagnosis is rather perplexing to a young practitioner, I am induced to offer a few remarks upon an accident which, although comparatively rare, every surgeon is liable to be consulted upon. The first case that came under my notice happened soon after I left the hospitals, and I well remember how difficult it was to account for the symptoms, for I had been taught that partial transverse fracture was impossible. But I am convinced that a bone may be bent, and the convex portion of the curve be cracked, and yet the fracture be incomplete and unattended with loss of continuity, as a tough twig may by bending be partially broken, and remain permanently curved, although not disunited. In the following case, which occurred but a short time since, the symptoms, peculiar to this injury, were well marked.

A fine, stout, ruddy boy, five years of age, son of B. Warren, Esq., of Clapham-park, was thrown from a donkey with considerable force; in falling he stretched out his left arm to save himself, and received a severe concussion on the ball of the left thumb. I saw him two hours after the accident; the palm of the hand was contused, but the principal injury was at the middle of the forearm which was swollen and bent, presenting the appearance of a transverse fracture of the radius and ulna. It was easy to ascertain that there was no dislocation, and that the ulna was uninjured; the head of the radius could be distinctly felt to rotate upon moving the wrist: but this bone was bent, the convexity of the curve being on the external aspect, and there was a corresponding hollow on the ulnar plane: there was no crepitus. Extension made no change in the appearance of the limb. The bone seemed to have been forcibly bent by the approximation of its distal and proximal extremities, occasioned by the violence of the concussion on the ball of the thumb, having produced partial fracture through the convex or bowed part, but not extending across the shaft, so as to occasion a loss of continuity. Leeches and the customary treatment were had recourse to; at the expiration of a week the swelling had subsided, but the curvature remained. In a few weeks after the accident the child could use the limb without inconvenience, and the deformity gradually disappeared. The other cases were attended with similar results: in none did I succeed in altering the bent condition of the bone, although extension was used carefully, and in some instances immediately after the accident; in all, the arm was ultimately restored to its normal state by proper exercise after the inflammatory symptoms had subsided. In every instance the *radius* was the bone fractured, and the patients were under nine years of age. In accidents of this nature, I would suggest that some attempts to remove the curvature by extension must be highly injurious, if sufficiently powerful to be effective; the application of leeches and the antiphlogistic means should alone be employed, for the action of the muscles will ultimately restore the limb to its natural form.—*Lancet.*

We have quoted the above extract for the express purpose of making it the subject of some comments.

The bending of the bones of children was formerly disbelieved by eminent surgeons in this country. The late Dr. Physick positively denied the possibility of such an accident, until a case of this character was presented to his observation by Dr. John Rhea Barton, who was the first to call attention to the subject. By one of those singular trains of events which, though seemingly fortuitous, conflict with all ordinary laws of chance, a number of cases came under his notice, in the course of a few years, at the Pennsylvania Hospital and elsewhere, and the current did not cease until long after we succeeded him as a resident surgeon of that institution. The paper of Dr. Barton, and several observations of our own in lectures and reviews, rendered the knowledge of this class of accidents very general some fifteen years ago; but, from the absence of further comments in the journals, it is fairly inferable that such cases are rare, numerous as were those presented prior to that time. We have met with but one case within the last twelve years!

The present Professor of Surgery in the University of Pennsylvania then insisted upon the existence of partial fracture in every instance, but in most of our cases, which could not have been less than ten or twelve in number, no evidences of fracture existed.

The restitution of the natural form of the limb by muscular action in the cases presented to Dr. Mantel is exceedingly singular, and had we trusted to the probability of such a result in ours, we should have suffered certain disappointment. The forearm, in several instances, was bent into the form of the letter S, and the curvatures were so great that their removal by the muscles was absolutely impossible. Nor would the stiffness of the bones have admitted of such a result, even if the direction of the muscular forces had favoured it. That osseous fibres in the interior were fractured, is extremely probable; for otherwise it is difficult to understand how substances so elastic as the chondroid and calcareous deposits could have retained their curvature: but that an actual fissure existed in most of these cases, we do not believe. By very firm extension, and by bending the bones in a direction opposite to their curvature, the limbs were restored perfectly to their natural form in every instance, with one exception. This was a case of decided partial fracture of both bones of the forearm, occurring in a boy at the age of about six years. The deformity was seated about two inches above the wrist joint, where the bones formed a very considerable and abrupt angle forwards. The sharpness of the projection at this point clearly showed that the bones were not simply bent, as usual in such accidents; but the resistance to extension as plainly proved that there existed no complete fracture. Decidedly, in this case, the muscles could not have restored the proper position of the bones. We were compelled to apply very considerable force in the reduction, and before it was quite consummated, the remainder of the diameters of the two bones gave way—the only case in which the fracture has been rendered complete by the surgeon that has occurred within the range of our own observation, though we have heard of other instances. The four extremities being all somewhat bent by the accident, a slight deformity remained after the reduction, but it was fortunately too trivial to attract attention or embarrass motion. Whether it ever disappeared with the growth of the patient, who, if living, must be now almost twenty-five years of age, we know not. He was an out-door patient, resorting to the Pennsylvania Hospital as to a Dispensary.

R. C.

---

In many cases of diseased heart there is a dropsical effusion, without any great obstruction at the valves. In the large majority of such cases, however, this obstruction exists; hence it is the most frequent, though not the only cause. In fact, dropsy evidently depends upon several morbid states of the heart, and upon a serous or watery state of the blood, which favours its development in many cases when the heart affection is not in itself sufficient for its production. Dropsy is, therefore, much more frequent in advanced cases of heart disease, not only because the lesion is more considerable, but because the blood is necessarily more and more apt to furnish its serous constituents.

As to the heart itself, we may say in general terms that some obstruction to the circulation exists. This may be of an acute kind, and dependent upon a mere inflammation which thickens the blood, favours the formation of fibrine, and the thickening or vegetations of the valves. This is the dropsy which occurs so frequently in endocarditis, and it is doubly apt to occur when the inflammation is grafted upon another cause or pre-existent lesion. Secondly, dropsy may arise from thickening and contraction of the semilunar valves, especially of the aorta, or even from dilatation of the heart when the aortic orifice remains of the natural size, and consequently too small for the easy passage of the large quantity of blood contained in the dilated ventricle; or, lastly, the effusion may depend mainly upon regurgitation of the blood from defective closure of a valve. One variety of this kind is the one alluded to by Dr. Blakiston. In a therapeutic view, it is of considerable importance to distinguish dropsy dependent upon defective closure of a valve with dilatation of the heart, because if the organ is much enfeebled by undue depletion, its power of impulsion may not be sufficient to relieve it of the accumulation of blood, and dropsy is more apt to ensue. These cases should be treated by moderate cupping, and small doses of digitalis combined with assafœtida, camphor, or Hoffman's anodyne. This acts as a regulator of the heart's action, without greatly enfeebling it.

We give the most important of the remarks of Dr. Blakiston on this subject:

As, indeed, dilatation of the heart, with or without hypertrophy, is, of all others, the most constant alteration of this organ, co-existing with cardiac dropsy, it is highly probable that some additional obstacle to the circulation is somehow connected with it, if not dependent upon it: and as the most direct obstruction to the venous circulation must exist on the right side of the heart, it is still further probable that it is connected with dilatation of that side.

Now the dilatation of the right ventricle necessarily gives rise to the enlargement of the tricuspid foramen, unless in such cases where the fibrinous zone, from which its valves spring, shall have to a certain extent lost its natural elasticity. One such case I have seen. The tricuspid valves intended to effect the closure of this aperture during the contraction of the ventricle, are only just sufficient for this purpose. Hunter, and, at a later period, Dr. Adams and Mr. King, have observed that they do not very effectually close the orifice they are attached to in its healthy state. Consequently, if this orifice become dilated, the valves can no longer effect its closure, except they also increase in size. In some cases of hypertrophy such is the case, but I think not so generally as Dr. Hope supposes; at least, as far as my own observations, confirmed by those of Bouillaud, would lead me to suppose.

Hence, regurgitation must not unfrequently take place through the tricuspid foramen during the systole of a dilated right ventricle; and thus a most powerful obstacle is opposed to the venous current, by a quantity of blood being constantly forced back upon it: and although this may in certain cases act as a safety-valve to the lungs, as supposed by Mr. King, yet if at all extensive and continuous, it can hardly fail to offer a most effective obstruction to the circulation returning from the system. This regurgitation through the tricuspid foramen is a frequent and direct cause of cardiac dropsy. The same obstruction would be produced by any other causes which prevent the tricuspid valves from closing the foramen to which they are attached, whether dilated or not. There are two causes of this sort which have hardly received the attention they deserve. The one is a shortening and thickening of the cordæ tendiniæ, which Mr. Hodgson tells me he has frequently seen; and the other, a partial or total adherence of the valves to the walls of the ventricle, unaccompanied by any other traces of disease.

A difference of opinion seems to exist between writers on diseases of the heart, as to the sufficiency of the proofs of regurgitation through the tricuspid foramen, both as drawn from observation during life and autopsy after death. Thus pulsation of the jugular veins, synchronous or nearly so with the systole of the ventricles, which have been considered by most writers as indicative of such regurgitation, are looked upon by Dr. Hope, as produced by the force of the right ventricular systole, and altogether independent of the completeness or incompleteness of the tricuspid valves.

It must be borne in mind, however, that considerable hypertrophy of the right ventricle is not unfrequently found in the bodies of persons who during life-time presented no appearance of jugular pulsation. And were it possible that the ventricle could ever contract with such force as to communicate a shock to the valves and blood above them, which should run up the jugular veins, it could only be in cases of extreme hypertrophy of the right ventricle, and therefore would be or rare occurrence, and would be accompanied by signs of such hypertrophy.

When, therefore, pulsations or obscure fluctuation of the jugular veins are observed, unaccompanied by any very strong heaving impulse of the heart, it is, I think, highly probable that regurgitation takes place through the tricuspid foramen. A perusal of the cases brought forward in this paper will be found very much to favour this view, inasmuch as in all those where venous pulsations or undulations were observed during life-time, the tricuspid foramen was found incomplete, either from its dilatation or the imperfect action of its valves, or from both causes.

That the force of pulsation of the jugular veins is a measure of the power of the right ventricle, and not a measure of the degree of regurgitation and obstruction to the circulation, I readily admit; for in all cases the stronger be the force of the right ventricle which throws the blood back upon the veins, the stronger will be the pulsation propagated up them. Not so as regards the obstruction to the circulation, which depends on the relative proportion between the power of the two ventricles. If both be hypertrophied, the pulsation or shock will be great, and if both be attenuated, the pulsation will be weak, while the obstruction to the circulation is the same in each case; for the venous current, and that of the regurgitating fluid, being both mainly derived from ventricular contraction, when the one is strong it is opposed by the other equally strong; and when one is weak, by the other equally weak; the obstruction is therefore the same in each case, and it is perfectly independent of the shock or pulsation.

If the proportion between the power of the two ventricles be altered, the effect on the circulation is altered; thus, if the right ventricle be hypertrophied and the left attenuated, a weak venous current is opposed by a powerful current of regurgitation, and the circulation is powerfully obstructed; if, on the other hand, the left ventricle be hypertrophied and the right attenuated, a strong venous current is opposed by a weak current of regurgitation, and a feeble resistance is offered to the circulation.

While, therefore, pulsations of the jugular veins may, in certain cases, be considered as indicative of regurgitation through the tricuspid foramen, their absence affords no proof of the non-existence of such regurgitation.

It has been proposed to test the completeness of the tricuspid valves after death by the manner in which they retain fluid in the right ventricle, which has either been forced or injected into it through the pulmonary artery. Mr. King has seldom found the valves to retain fluid when the experiment has been made with a healthy heart; and I have several times repeated the experiment with no better success, unless I pinched in the base of the right ventricle surrounding the foramen with my hand. This, therefore, would be an unfair test, as it would go to prove that almost all tricuspid valves were incomplete. Bouillaud has compared the measure of the circumference of the foramen with the height of the valves from their apex to the middle of their base. This latter plan gives a very insufficient idea of the area the valves can cover; because unless the circumference of the foramen be retained in its proper position, which is very difficult to do, the valves will be stretched, and will measure more than they can do in action.

He gives three inches eleven lines as the mean circumference of an undilated tricuspid foramen.

I have been in the habit of removing the apex of the heart and laying open the right auricle, and then raising up the tricuspid valves into their plane of closure, by means of my fingers introduced into the right ventricle. By looking at them from the auricle when in this position, a tolerably accurate idea can be formed of the area they cover, and whether any and how much space is left uncovered by them, through which the blood can regurgitate. I have then usually guaged the tricuspid foramen by the introduction of the fingers; its ordinary size not quite admitting the three first fingers up to their first joint, and then measured the circumference after it has been laid open. I regret that the size of the heart should have been noted in such loose terms. I have lately measured its bulk and capacity, by ascertaining the quantity of fluid it displaces both when full and empty, and hope by this means to arrive at a more accurate standard of measurement.—*Lond. Med. Gaz.*

---

*On the Employment of Cold Affusion in the Treatment of Acute Hyrdocephalus.* By Dr. Münchmeyer, of Verden.—Dr. Münchmeyer observes that the medical world is greatly divided in opinion as to the value of this remedy; some persons greatly extolling its efficacy, while others regard it as altogether useless. He considers it to be a most important remedy, and one which will often save life when all other means have been useless. One great reason why cold affusion has met with so few supporters is to be found in the misconception which has prevailed with reference to the proper time for using it. It is certainly not always advisable to resort to it, and it should never be forgotten that its mode of action differs essentially from that of cold when kept constantly applied to the head. In the employment of cold affusion it is the secondary action of cold, as well as the sudden shock to the system produced by the mode of its application from which benefit is expected, while in the case of cold lotions to the head it is the primary action of cold which is obtained. Cold affusion then must not be looked upon as a directly antiphlogistic remedy, nor is its employment indicated during the early inflammatory stages of hydrocephalus, but rather when effusion, the consequence of inflammatory action has taken place, and a tendency to paralysis exists. After the subsidence of the violent symptoms of the disease, and when the patient has sunk into a comatose state, with a pale countenance occasionally suffused with a flush, dilated pupils, strabismus, and slow pulse, this remedy will frequently prove of excellent service.

In order, however, for benefit to be derived from it, it must be employed in an efficient manner. Dr. Münchmeyer directs that the patient should be taken out of bed, stripped of his clothes, and wrapped up in some simple covering, (if waterproof the better,) which leaves only his head exposed. He should then be placed in a sitting posture in a bath or tub, and the person who administers the affusion should mount upon a chair and pour cold water upon his head, in a moderate stream from the height of five or six feet. This may be continued for a minute or two, and repeated twice or thrice. The patient should then be wrapped up in a warm sheet and placed in bed, where he should remain till it is thought proper again to have recourse to the remedy. At first, it will probably be requisite to repeat the affusion in the course of an hour and a half or two hours; but as the patient improves the interval may be longer, so that at last it will not be necessary to employ it above two or three times daily.

The immediate effect of cold affusion is, that the patients awake from their comatose condition and begin to cry violently, which they continue to do so long as the water is poured upon them. They afterwards appear exhausted and pale, the skin is cool, the pulse small and very frequent. When placed in bed they usually fall into a dose, the pulse becomes more regular, and the warmth of the skin returns. By degrees as with the repetition of the remedy the patients improve, they begin to have sound sleep, from which they awake in the possession of all their senses, recognize those by whom they are surrounded, and cease to squint. At the same time, too, a sweat, frequently of a critical nature, breaks out upon the whole body, and during its continuance the employment of cold affusion is very hazardous. The patient's sleep becomes more refreshing, and the comatose condition recurs at longer intervals; he begins to notice what goes on around him, the head regains its natural temperature, and the febrile symptoms disappear. The employment of affusion must, however, still be continued for some days, since relapses very frequently occur.

The paper is illustrated by five cases. In three, the employment of cold affusion was perfectly successful; in one, it produced temporary amendment, and the death of the patient was to all appearance owing to the apathy of the parents, who neglected to persevere in the treatment; while in the fifth, convulsions and death followed affusion while the patient was perspiring profusely.—*Brit. and For. Med. Rev., from Hannoversche Annalen,* Band v. Heft 4.

We do not agree with the author as to the advantages of the cold affusion in acute hydrocephalus, which is essentially an inflammatory disease, and, when once arrived at the period of coma, almost necessarily fatal. But in comatose affections of a different kind, depending upon congestion of the brain, or even upon a nervous stupor (for such there is) without any vascular lesion, it is highly valuable.

---

We make no apology for the length of the following extract, with the accompanying comments of the British Reviewer, for the case is one of great interest in its bearing upon the value of circumstantial evidence in trials for murder. Carelessness in questions of a medico-legal character saddles society with the continued presence of many of the worst criminals, while there can be little doubt that it sometimes leads to the capital condemnation of the innocent.

*Case of Death by Hanging, with numerous Wounds on the Person.* By Dr. KRÜGELSTEIN, of Ohrdruff.—The deceased was about forty-eight years old; she had borne several children, and enjoyed good health and spirits up to the time of her last accouchment. She then became morose and unhappy, and attempted to drown herself. One morning she was found hanging, quite dead, in a cowshed adjoining her bed-room.

On examining the room in which she slept, some coagulated blood was found near the bed on the floor, and there were marks of bloody fingers on the walls, but there was no trace of blood on the bed itself. From this room to the shed in which her body was found, there were no traces of blood, but there were impressions of bloody hands on the walls of the shed; and the beam from which her body was suspended, as well as the cord, was slightly stained. On the floor beneath the body there were marks of the fæces having been discharged. The roof of the shed was low, but sufficiently high to allow a person to be freely suspended from the floor. There was some dried blood on the face and dress.

The following appearances were met with:

On the tuberosity of the os occipitis, a severe contusion of a livid colour and about two inches in circumference. On cutting into it, coagulated blood was found beneath, but the skull was uninjured. Over the left temple there were seventeen incised wounds about two or three inches long, some penetrating to the bone, but none involving the temporal artery. On the left side of the os occipitis were two superficial cuts, and on the upper part of the right and left parietal bones were many others of a similar character. On the right temple there were so many deep incisions as to have completely destroyed the temporal muscle. Over the sagittal suture was a long wound penetrating to the bone.

The face was pale and free from any marks of violence; the eyelids were closed, and the eyes not projecting. The mouth was open; the tongue not prominent; there was no appearance of frothy mucus either in the mouth or nose.

Around the neck and above the larynx a tolerably deep depression had been produced by the cord. This extended beneath the angle of the jaw and the mastoid process on either side to the nape of the neck. Notwithstanding the cord had thus caused a deep and well-defined mark, there was no ecchymosis, if we except a slight bluish tint on the left side; nor had the skin acquired that parchment-like character which is often seen in the hanged. Neither the os hyoides, trachea, nor the vertebræ of the neck had sustained any mechanical injury.

The surface of the body was more or less covered with blood which had flowed from the wounds. Over the sternum and region of the stomach numerous but small incised wounds were perceived, which, from their appearance, had evidently been produced several days before those met with on the head. This was proved by the fact that some of them were passing into the suppurative stage.

On opening the cranium, splinters of the internal table were found corresponding to the wound in the region of the sagittal suture. These pressed on the dura mater, but had not injured it. There was no congestion, effusion, or injury to the brain, and the only morbid appearance was an hydatid cyst in one part of the pia mater. The lungs were of a bluish white colour, not particularly congested; a bloody froth escaped on making an incision into them. The heart and the abdominal viscera were in a normal state. The bladder was empty. The genitals were not turgid, as they are often described to be in females who have died by hanging.

The medico-legal questions to determine here were, as to whether: 1, the numerous wounds on the body had been self-inflicted; 2, whether the hanging was the result of suicide or murder.

The account given of the deceased showed that she had a predisposition to suicide. This is borne out medically by the discovery of hydatids in the pia mater.

In the shed where the body of the deceased was hanging, an axe was found with which she had no doubt produced the injuries to her head. The edge of the axe was sufficiently sharp to account for the production of the numerous incised wounds found in the scalp, and the weapon was long enough to permit her to have produced the *severe contusion found on the os occipitis.* Had such a weapon been used by a murderer, one or two wounds only, and those of a much more severe character, would have been produced. As it was there were no less than *fifty-five* wounds about the scalp, and most of these unimportant.

As negative proofs of this not being a homicidal act, may be mentioned: 1, that there was no mark of violence on the person, discomposure of the dress, or disturbance of the furniture in the room; 2, the act must have been perpetrated at a time when any noise or struggling would have been heard by the neighbours; but nothing of the kind was remarked.

The conclusions of the inspectors in this case were:

1. That the deceased did not die from the wounds found on her person nor from the loss of blood.

2. That death was the result of hanging, operating partly through pressure on the nerves and partly through suffocation.

3. That it was an act of suicide.

[Remarks. This case is in many points interesting to the medical jurist. Had it occurred in England, it is scarcely necessary to observe that a coroner's jury would, under the circumstances, have deemed an inspection of the body useless, and as only giving rise to unnecessary delay and expense. Yet it is by the accurate observation of cases of this kind that materials can be collected, which will enable a medical witness to prevent coroners' juries from falling into mistakes in future investigations where the circumstances are of a more doubtful character. Out of the hundreds of cases of suicidal hanging which occur in this country, how many bodies are inspected by the order of a coroner? Scarcely one per cent.! We do not believe that so complete and accurate an investigation of the subject of hanging could be produced from the records of coroners' inquests, although the opportunities for benefiting science by this kind of information have been numberless.

This case possesses more than usual interest: 1, On account of the great number of wounds found on the head; 2, from the *nature* of some of the wounds; thus those over the sagittal suture were sufficient to cause a separation of the inner table of the skull: 3, from the situation. One violent contusion was seen on the os occipitis, a most unusual situation for a suicidal injury. It is remarkable that from the two last-mentioned circumstances concussion of the brain, depriving the person of the power of subsequently hanging herself, had not resulted, since there was evidently violence enough to account for an immediate loss of sense and motion. On the whole, suicide seems to have been more established from *general* than from *medical* facts; and had the deceased been found thus suspended in an exposed place, a strong suspicion of murder might fairly have arisen in the minds of the inspectors.]—*Ibid., from Henke Zeitschrift für des Staatsarzneikunde.* i. 1840.

---

*Direct Experiments to determine whether portions cut off from Leeches are reproduced.* By Stefano Grandoni.—Bose, by whom Buffon's Natural History was continued, positively asserts the reproduction of divided leeches; in the Dictionnaire des Sciences Naturelles the contrary is stated. This contradiction with other circumstances induced the author to submit the question to the test of experiment. He divided twenty leeches between the fifth and sixth segments of their bodies, and placed half of them in glasses containing water with little stones at the bottom, and the other half in glasses containing a thin stratum of clay moistened with water. All possible care was taken to maintain them in health. After three weeks, one of those that lived in the clay exhibited on its truncated extremity two white and somewhat convex gelatinous corpuscles, in the centre of which there was a more vividly colored and transparent point; the rest of the section was somewhat rounded, and covered by a very delicate membrane. These signs seemed to render it probable that the experiment was about to succeed; but a month after the leech died, and apparently from the disease of the truncated extremity. Three months after being divided, the sections of the other leeches were found covered by a dense round gelatinous mass, which, after some time, became a very fine and transparent investment, and made the surface of the divided part quite smooth. After about six months, the leeches in the water with the pebbles all died one after the other, without any regeneration of the part that had been cut away. Their weight was neither diminished nor increased. At this time, six of the remaining leeches were taken from the clay, in which they had till lately constantly lain buried, and put in water. These, as well as the three that were left in the clay, were then looked at several times every day; the condition of their trunks was from time to time examined with the aid of powerful lenses, and the number of their segments was counted, but no increase was observed. At the tenth month from the commencement of the experiment, only the three leeches in the clay remained alive; these also died four months afterwards, but without the reproduction of even the most minute ring. It was thus decided that divided leeches are not regenerated.—*Brit. and For. Med. Rev., from Com. dell' Ateneo di Brescia* 1837–8.

The question of the reproduction of parts in the leech becomes a question of greater interest to the general physiologist, from the fact that the relation between this reproductive power in animals, and the degree of complexity in the organization of the nervous system, is a matter of high interest in the discussion of elementary physiological principles in the present condition of the science. Well ordered experiments on this question would be peculiarly important at present; for, although the question may be regarded as nearly settled, it is well known that, in the kindred genus of planaria, the reproductive power is remarkably developed. This investigation would furnish a fine field of research for an ambitious and pains taking student, who wishes to distinguish himself by originality in a thesis. We should be happy to communicate some ideas to any such aspirant who may be disposed to undertake the investigation.

R. C.

---

*Report on M. Louvrier's Treatment of Anchylosis by sudden and forcible Extension.* By MM. Thillage and Berard.—The following is the substance of the above lengthy report. M. Louvrier's machine has been employed on 22 patients, of whom only three have experienced ill effects, all the others having escaped injury. Most of the patients suffered excessive pain at the moment of operation. In no case has the anchylosed articulation recovered entire freedom of motion. Those patients most successfully treated are obliged to use a staff in walking; one only walks without a stick, but the restraint is evident.

With regard to the unfortunate cases: in one female, in whom the anchylosis of the knee was complete, and the limb fully flexed, the application of the machine was followed by a very considerable rupture of the skin, luxation of the leg upon the posterior part of the thigh, and abundant suppuration, which terminated in death three weeks after the operation. At the necropsy, the articular cavity was found full of pus, the popliteal artery intact, the popliteal vein full of pus, and its coats thickened. Many muscles were ruptured and softened; the anterior crucial ligaments softened; one of the posterior softened, the other ruptured, attached by one extremity to the tibia, and terminating at its free extremity by an osseous portion, which was evidently part of the condyle of the femur, fractured at the moment of operation.

Another patient suffered excessively acute pains at the moment of operation, and remained some time in a sort of delirium occasioned by the suffering. Gangrene commenced on the next day, but was limited by the efforts of nature alone, and the patient is actually cured.

In a third case, that of a young woman in whom the anchylosed limb was fixed at a right angle, the straightening was incomplete. M. Louvrier applied a piece of wood to the anterior part of the knee, by means of which he hoped to press the limb into its natural position; but an eschar formed on the next day, and the patient died in six weeks.

In another patient who died from other causes, the articular extremity of the tibia was found to be luxated upon the posterior part of the femur, the internal condyle of the latter being fractured.

The number of these accidents, however, being small, [!] the opinion of the reporters would be less unfavourable, were the ill effects balanced by real advantages; but as the limb after operation is as immoveable as an artificial support, they conclude:

1. That the application of the machine of M. Louvrier is followed by an instantaneous straightening (*redressement*) of the anchylosed limb.
2. That it is not ordinarily followed by any severe symptom, primary or consecutive.
3. That when these accidents are produced, they are frightfully severe, and are ordinarily followed by death.
4. That none of the patients operated upon by this method have entirely recovered the free motions of the anchylosed articulation. We therefore report to the minister that the machine of M. Louvrier, although ingenious, is dangerous in application, for it will be always impossible to determine the nature of the anchylosis, and to foresee the conditions which would offer some chances of success for its employment.—*Ib., from Bull. de l'Acad. Roy. de Med.*

The madness of recent pseudo-surgery, and its recklessness of life and pain, was never more outrageously exemplified than in the horrible apparatus of M. Louvrier, and its actual application in cases of anchylosis. We are not particularly nervous about the use of forces that are in any degree warrantable, having re-fractured several limbs across our knee after the lapse of five or six weeks from the time of the accident, in order to remove deformities resulting from carelessness and ignorance in the early treatment of the cases; but the disruption of a joint, by mechanical power, in old anchylosis, is a thing not to be even contemplated with patience. We are astonished that the Academy should have gravely entertained a report on the subject. If an attempt to imitate the process should ever be made on this side of the Atlantic, it may be hoped that the folly may be confined to the brotherhood of natural bone-setters, and that the *courts of law* may take the proper steps on each occasion!

In false anchyloses, even when motion in the joint is almost imperceptible, we have seen enough, of late, to show that the straightening of the limb, or its flexion, if necessary, by means of moderate and permanent mechanical action, *is never hopeless.* When the bones have become amalgamated by the removal or ossification of all the intervening tissues, the operation by means of the saw, as first practised by Dr. John Rhea Barton, though it does not promise the restitution of the old joint nor the substitution of a new one, is far more defensible, terrible as it is. We are glad to perceive that the report of the MM. Thillage and Berard is calculated to give the death-blow to the operation, instead of the patient. R. C.

---

*Successful Case of Excision of the Head of the Humerus, after Compound Fracture.* By Dr. Hello, of Cherbourg.—An English boy, fifteen years of age, fell from a height on the 16th of April, 1841, and sustained a fracture of the humerus at the situation of its surgical neck. The lower fragment tore the integuments between the great pectoral and deltoid muscles, and appeared through the clothes. There was considerable hæmorrhage at the moment of fracture, but the fracture was almost immediately reduced, and the bleeding ceased. On passing the finger into the wound, it was found full of clots; the inferior fragment of the humerus was denuded of its periosteum to the extent of about six centimetres; the superior fragment, very short, offered a number of small splinters, which were almost attached to the articular capsule. I at once determined to lay bare the shoulder-joint, with the double purpose of resecting the humerus and extracting the head of the bone. A semicircular incision was made, commencing a little below the coracoid process, following the line which separates the great pectoral and deltoid, turning backwards at the insertion of the latter, which was cut to the bone, and remounting to the acromion along the external border of the deltoid. The flap thus formed was turned upwards, and the arteries nourishing it tied. The superior part of the humerus was then carried from the wound, (which was easily done by seizing the condyles to direct the bone,) the periosteum surrounded with the bistoury below the situation where it was torn, and after having placed a piece of pasteboard between the flesh and the bone, the denuded portion of the latter was removed by an ordinary saw. The posterior circumflex artery, which bled freely, was tied; and being cut so near the axillary trunk, it is not improbable that this might render secondary hæmorrhage frequent after this operation, the formation of the clot being prevented. The axillary artery was so superficial at the bottom of the wound, that I should have passed the ligature around it, would not this have hazarded the success of the means employed to save the limb. The musculo-cutaneous nerve which had been torn was excised, the flap reapplied, three sutures inserted, and the parts were then kept in apposition by adhesive plaster, and covered with compresses of charpie, the whole being maintained by a bandage crossing under the opposite axilla. The forearm and hand were placed in a sling, and the arm was separated from the chest by a soft cushion of charpie. The patient went on without a bad symptom until the 19th, when there was some fever, with fetid and abundant suppuration. On the 23d there was less fever, aud the suppuration more healthy. Two ligatures came away. Sutures removed. Internal part of wound cicatrized. All the ligatures had come away by the 29th. On the 1st of May a well-marked intermittent set in, for which quinine was given, and continued for several days. The wound had perfectly cicatrized by the 31st. The patient could seize small objects with the hand, and, having recovered his health, wished to return to England immediately.

This case is a very interesting one, showing that, under very favourable circumstances, a useful limb can be secured to the sufferer, and pointing out a very easy mode of performing the operation. The author states that he followed the same proceeding on a Turk at Marseilles; but he was sent to Smyrna on the fifth day, and the result has not been ascertained.—*Ib., from Ann. de la Chirurg.*

We know not what some of our friends of Cortland county may say to the practice adopted in this case, they being so strenuously opposed to the excision of bone. Nor are we altogether prepared to endorse the propriety of so serious an operation as that described above, without further details than are given in the outline of the case. Denudation of bone in a young lad of good constitution, must be carried very far before it becomes certainly a cause of serious necrosis, especially when the injury is located near the extremity of a long bone, where the vital activity of the osseous tissue is great. Near the head of the humerus, so close is the proximity to the heart, that this vital activity is displayed with remarkable energy. Nor is the splintering of a bone in compound fracture usually a sufficient cause for the excision of a joint,—*in civil practice*,—unless great displacement occurs, or the spiculæ are coated entirely with ligamentous matter, which limits the supply of blood to the fragments. We mean not to utter a condemnation of the operation, for the proper course of treatment in such cases often depends upon circumstances not very easily described in words; but, having spoken decidedly in favour of the excision of an inch of the tibia in our last number, under less equivocal circumstances, in a case of alleged mal-practice, it is not amiss to temper that opinion with a caution against too hasty intermeddling with fragments in compound fractures generally. It is fortunate that Dr. Hello did not tie the axillary artery. There would have been but doubtful excuse for so doing; and it is rather surprising that the thought should have been suggested. R. C.

*On the Temperature of Marine Invertebrata.* By VALENTIN and WILL.—The authors have made observations on the proper temperature of seventeen kinds of marine invertebrata, comprising Polyps, Medusæ, Echinodermata, Helices, Cephalopods, and Crustacea. They found that all of them had a peculiar temperature varying with, but always somewhat surpassing, that of the medium in which they lived. The greatest difference amounted to 1°, the least to 0.1°; the former was observed in Pelagia denticulata, the latter in Aplysia leporina. With regard to the several classes the differences were among

| | |
|---|---|
| Polyps, on an average | +0.205 |
| Medusæ | +0.27 |
| Echinodermata | +0.40 |
| Helices | +0.46 |
| Cephalopods | +0.57 |
| Crustacea | +0.60 |

Which numbers prove an increase of proper temperature directly proportioned to the ascent in position in the animal kingdom.—*Ib., from Müller's Archiv.*

*On Spontaneous Combustion of the Human Body.* By Dr. JACOBS, of Eupen.—From 28 cases of spontaneous combustion collected and analysed by the author, he concludes:

1. That spontaneous combustion always occurs in living human beings, never after death nor in the lower animals. 2. The subjects were generally very old, the two youngest being fifty and twenty-nine years of age. 3. Women are most frequently the subjects, it having only occurred it *two* men. 4. It was once preceded by jaundice, once by a malignant ulcer on the head. 5. All the persons were alone at the time of the occurrence. 6. They led an idle life. 7. All were very fat, except three very lean females. 8. Almost all were intemperate. 9. Most frequently a light or some ignited substance was in the neighbourhood at the time of the accident. 10. The combustion proceeds very rapidly, and finishes in seven, three, and two hours, and even in one hour. 11. The flame, difficult to be extinguished by water, was very mobile, only destroying the objects placed very near, or in immediate contact with the burning body.—12. The room in which the combustion took place was usually filled with a thick vapour, and the walls covered by a black carbonaceous substance; the floor, ashes, and bones imbued with fat and fetid moisture. 13. The trunk was most frequently completely destroyed, some parts of the head and extremities usually remaining. 14. This combustion has occurred, with only two exceptions, during a cold temperature, in winter, and in the northern regions.—*Ibid, from Bul. Gén. de Thérapeutique.*

*Injections of the Iris.* By Professor GRIMELLI, of Modena.—The author has made some experiments to support the opinions of Dr. Fario upon the vascular erectile structure of the iris. The substances which answer best for very fine injections of this organ, are olive or walnut oil variously coloured, which penetrates into the most delicate vascular ramusculi without transuding through their coats, and preserves, for a long time, the parts which they impregnate. In injecting the bodies of children, Dr. Grimelli observed, that from being soft and much dilated, the iris became turgid, and contracted more than half its diameter, in the same manner as when the retina is affected by light during life. This fact appears to prove that the iris is composed of a union of vessels forming a disc, in the centre of which is the pupillary aperture, and the circumference of which is attached to the ciliary ligament. By the aid of the lens and microscope, we see that the very fine vessels which constitute the iris are disposed between the pupillary and ciliary circles, under the form of rectilinear and curvilinear radii, curved upon themselves and zigzag; agglomerated and united in an inextricable manner. We observe also some ramifications disposed in circles between the pupillary and ciliary circles, more or less near each other, and always few in number. It results from this disposition of the minute vessels, fixed towards the larger circle and moveable towards the lesser, that the sanguineous afflux and turgescence unfold the iris and contract the pupil, and that the return of blood and diminution of turgescence, fold again or wrinkle the membrane and dilate the pupillary aperture. Thus, contrary to the generally admitted opinion on the muscularity of the iris, as it appears to the author, this membrane is composed of a turgescible or erectile vascular tissue, in which arterial vessels predominate, and Dr. Grimelli is led by analogy to conclude that the muscles of the small bones of the ear are constituted in the same manner.—*Ibid., from Revue Médicale.*

